# Gradient Variation and Correlation Analysis of Physical and Mechanical Properties of Moso Bamboo (*Phyllostachys edulis*)

**DOI:** 10.3390/ma17092069

**Published:** 2024-04-28

**Authors:** Tian Jiang, Xinyu Feng, Zexuan Xia, Shuotong Deng, Xuehua Wang

**Affiliations:** College of Furnishings and Industrial Design, Nanjing Forestry University, Nanjing 210037, China

**Keywords:** moso bamboo, vascular bundle, chemical content, bending strength, linear correlation

## Abstract

This study aimed to investigate the gradient properties of bamboo at the microscopic level and provide a basis for improving the utilization rate of bamboo. Using moso bamboo (*Phyllostachys edulis* (Carrière) J. Houz.) as a research subject, the variation of vascular bundle area percentage, chemical content, relative crystallinity (CR), mechanical properties of different bamboo slivers, and correlation between those parameters were analyzed. From the bamboo green layer (BGL) to the bamboo yellow layer (BYL), the distribution of vascular bundles changed from dense to sparse. Cellulose and lignin mass content decreased gently, and hemicellulose mass content showed gradual increases. The CR showed an order of bamboo middle layer (BML) > BGL > BYL. The tensile modulus of elasticity, tensile strength, bending modulus of elasticity, and bending strength decreased from BGL to BYL. The order of influence degree on mechanical properties of moso bamboo was vascular bundle area, hemicellulose content, lignin mass content, density, and CR, and these factors correlated with mechanical properties at a significant level (*p <* 0.05). Vascular bundle area had a decisive effect on the mechanical properties of bamboo. The vascular bundle area and density were linearly correlated with mechanical properties, while the lignin mass content and CR were curve-linearly correlated with mechanical properties.

## 1. Introduction

Bamboo is a promising alternative resource for wood [1], garnering significant attention for its remarkable mechanical properties [2]. According to research, the longitudinal stiffness of bamboo is similar to that of wood, with a tensile strength about twice that of wood and a specific strength approximately 2–3 times that of steel. Moreover, the fracture toughness and fatigue performance of bamboo exceed those of most engineering materials [3]. These superior mechanical properties are largely attributed to the unique gradient structure. The term “gradient structure“ refers to a transitional, non-uniform structure in which one structure, component, or phase gradually changes. Concurrently, its microscopic structure, physiochemical properties, and mechanical strength undergo stage-wise changes [4]. The bamboo tube, characterized by its hollowness, comprises a microstructure primarily consisting of vascular bundles and longitudinally arranged parenchyma cells [5]. Along the radial direction of the bamboo wall, the density of vascular bundle distribution decreased from the bamboo green layer (BGL) to the bamboo yellow layer (BYL), displaying a distinct gradient distribution. This gradient structure of bamboo has evolved through continuous optimization processes and is widely acknowledged for its role in enhancing mechanical properties. Wei [6] found that the gradient distribution of fibers is crucial for variations in the bending resistance of bamboo, and the asymmetric distribution of bamboo fibers contributes to variations in its bending properties along different directions. The gradient distribution of vascular bundles plays a crucial role in determining the variation of their chemical composition and mechanical properties. Tommy [7] concluded that fiber distribution density on bamboo cross-section is a significant factor influencing the physical properties of bamboo. Moreover, the uneven distribution of parenchyma cells and fibers constitutes an essential reason for the gradient structure and its effects on its strength and toughness [8]. Huang et al. [9] found that the gradient variation in bamboo structure, such as density, matches the mechanical property, such as bending strength, of its corresponding position. Wang et al. [10] found that the tensile strength decreases as the moisture content decreases, while the tensile modulus is relatively unaffected by changes in moisture content. Zhou et al. [11] analyzed the relationship between the mechanical properties of bamboo and its density and confirmed that there is a strong correlation between them. Liu et al. [12] found through correlation analysis that strip harvesting can reduce the harvesting time and costs of moso bamboo forests. However, different harvesting widths will affect the physical and mechanical properties of moso bamboo to varying degrees. Yu [13] found that the relative density, tangential shrinkage, tensile modulus of elasticity, and tensile strength of bamboo increase from the inner layer to the outer layer, while longitudinal shrinkage decreases. Dixon et al. [14] found that the gradient change in radial and longitudinal density of moso bamboo is a significant factor leading to variations in mechanical properties. Rodolfo et al. [15] utilized robotic fabrication to determine the physical and mechanical properties of bamboo and found that when considering only the mean values of each bamboo attribute, there is a relatively strong correlation between the average mechanical performance and density and volume fraction.

Currently, research on the gradient structure and properties of bamboo mainly focuses on the physical or mechanical properties along the radial direction of bamboo. However, there is a lack of systematic research on the correlation between physical and mechanical properties and microstructure, and the parameters of this correlation are not yet clear. Due to the gradient structure of bamboo, there are significant differences in the properties of BGL, BYL, and bamboo middle layer (BML). In production processes, it is common practice to remove BYL and BGL from the original bamboo, which ultimately leads to a low utilization rate of bamboo.

Therefore, moso bamboo was taken as a research object. The mechanical properties, including tensile strength, tensile modulus of elasticity, bending strength, and bending modulus of elasticity of bamboo strips and bamboo slivers along the radial direction from BGL to BYL were investigated using a mechanical testing machine. Additionally, measurements were taken on its chemical composition, relative crystallinity, and microstructure. The gradient change trend and correlation of the above parameters were analyzed. This study aims to provide references for the rational utilization of bamboo’s gradient structure, facilitating the high-value utilization of bamboo materials.

## 2. Material and Method

### 2.1. Material

**Figure 1:**
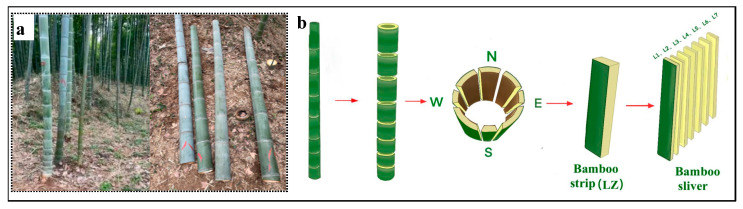
Sample preparation. (**a**) Sample and (**b**) bamboo sliver sample preparation.

Given the well-developed maturity of four-year-old moso bamboo (*Phyllostachys edulis* (Carrière) J. Houz.), its mechanical properties and physical characteristics remain stable. Hence, it was selected as the focal point of this study. Sourced from the bamboo forest base within the Purple Mountain Scenic Area, situated in the Xuanwu District of Nanjing City, Jiangsu Province, the material originates from a region characterized by a subtropical monsoon climate in China’s mid-latitude zone. The bamboo sections, selected for sample preparation, were gathered from 1.3 m to 3.3 m above the ground (Figure 1a). The average bamboo stem diameter was about 100 mm, and the bamboo wall thickness was 10 ± 2 mm. The internode portion of the bamboo culm was used and subsequently dried in an oven (Yiheng, Shanghai, China) at 40 °C until reaching a moisture content of about 10%. After drying, samples were extracted from each bamboo culm, oriented in the east, south, west, and north directions, according to the Chinese standard GB/T 15780-1995 [16]. These samples were then cut into bamboo strips, which were marked as LZ, all standardized to dimensions of 240 mm × 8 mm × (8 ± 0.5) mm (longitudinal × tangential × radial). Seven layers of bamboo slivers, approximately 1 mm thick, were prepared along the radial direction of the bamboo strip, denoted sequentially as L1 to L7, from BGL to BYL, respectively (Figure 1b).

The density of bamboo strips and slivers was assessed using an oven drying method. Eight parallel samples were prepared, and the arithmetic mean value was computed, with the standard deviation serving as error bars.

### 2.2. Mechanical Property Testing

**Figure 2:**
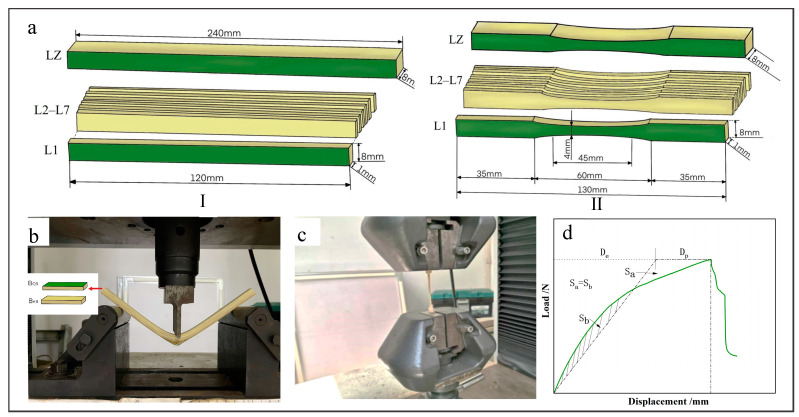
Mechanical property testing method. (**a**) Sample for bending test (**Ⅰ**) and tensile test (**Ⅱ**), (**b**) bending test, (**c**) tensile test, (**d**) plastic displacement test method.

The bending and tensile properties were obtained using the Instron Universal Mechanical Testing Machine (Instron, Boston, MA, USA). The dimensions of the bending samples were 120 mm× 8 mm × 8 mm for bamboo strip and 120 mm × 8 mm × 1 mm for bamboo sliver (Figure 2(aⅠ)). The tensile samples, which had a dumbbell shape, were cut by hand. The dimensions for the bamboo strip were 130 mm × 8 mm × 8 mm, and for the bamboo sliver, they were 130 mm × 8 mm × 1 mm (Figure 2(aⅡ)).

The three-point bending method was utilized for the bending test, with a span of 40 mm for bamboo sliver and 170 mm for bamboo strip (Figure 2b). The loading speed was 5 mm/s to ensure that samples failed within 1 ± 0.5 min. Two loading directions along the radial direction of bamboo samples were tested, of which the loading header on BGL was marked as B_QB_, while on BYL, it was marked as B_HB_. In the tensile test, the specimens were uniformly loaded at a speed of 5 mm/s to ensure that they failed within 1 ± 0.5 min (Figure 2c). Each group was set up with 8 parallel samples, and the arithmetic mean was calculated, with the standard deviation used as error bars. From these experiments, the bending strength ($\sigma_{b}$), bending elastic modulus ($E_{b}$), tensile strength ($\sigma_{t}$), and tensile elastic modulus ($E_{t}$) were obtained. The calculation formulas for these mechanical property indexes are as follows:

$$\sigma_{b}=\frac{3P_{max}L}{2bh^{2}}$$

where $\sigma_{b}$ is bending strength, MPa. $P_{max}$ is the failure load, N. L is the span between the supports, mm. b is the width of the test section in the specimen, mm.  h is the height of the test section in the specimen, mm.

$$E_{b}=\frac{{PL}^{3}}{{4bh}^{3}f}$$

where $E_{b}$ is bending modulus of elasticity, GPa. P is the load difference value between the upward and downward loads, N. L is the span between the supports, mm. b is the width of the test section in the specimen, mm. h is the height of the test section in the specimen, mm. f  is the deformation difference value of the specimen between the upward and downward loads, mm.

$$\sigma_{t}=\frac{P_{max}L}{bt}$$

where $\sigma_{t}$ is tensile strength, MPa. $P_{max}\;$ is the failure load, N. L is the span between the supports, mm. b is the width of the test section in the specimen, mm. t  is the thickness of the test section in the specimen, mm.

$$E_{t}=\frac{20∆P}{bt∆l}$$

where $E_{t}$ is tensile modulus of elasticity, MPa. ∆P is the load difference value between the upper and lower load limits, N. b is the width of the test section in the specimen, mm. t is the thickness of the test section in the specimen, mm. ∆l is the deformation difference value of the specimen under upper and lower load limits, mm.

Furthermore, the plastic displacement (Dp) was obtained from the load–displacement curve by the equivalent elasto-plastic energy method (Figure 2d) [17].

### 2.3. Microstructure and Chemical Characterization

Environmental scanning electron microscopy (Quanta200, FEI, Lexington, KY, USA) was employed to investigate the microstructure, with a focus on determining the proportion of vascular bundles based on SEM images. Bamboo samples, with a size of 10 mm × 2 mm × 8 mm (longitudinal × tangential × radial), were cut using a sliding slicer. The sample surfaces were manually smoothed using a blade. After air-drying, the samples were sprayed with a gold coating and scanned by a scanning electron microscope at a voltage of 15 kV to obtain a microscopic view of the cross-section.

The proportion of vascular bundle area was measured by the software ImageJ (1.53a, National Institutes of Health, Bethesda, MD, USA). The formula for calculating the proportion of vascular bundle area was:

$$p_{v}=\frac{S_{V}}{S_{Z}}\times 100\%$$

where, $p_{v}$ is the vascular bundle area percentage, %; $S_{V}$ is the vascular bundle area on the cross-section; $S_{Z}$ is the total area on bamboo cross-section.

**Figure 3:**
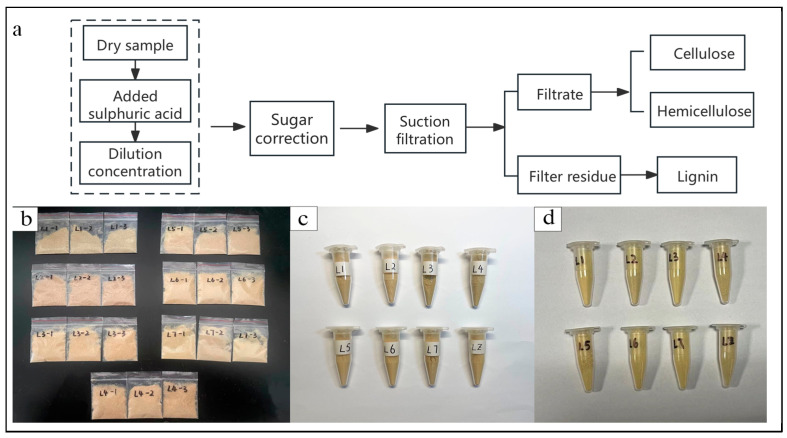
Chemical Performance Testing. (**a**) The process of the NREL method, (**b**) NREL samples, (**c**) XRD samples, and (**d**) FTIR samples.

The NREL (National Renewable Energy Laboratory) method was used to determine the content of cellulose, hemicellulose, and lignin in bamboo (Figure 3a) [18]. The bamboo slivers from L1 to L7 and bamboo strip were crushed by a pulverizer, then sieved, and bamboo powder between 100 mesh and 200 mesh was dried at 105 °C and tested according to the NREL method (Figure 3b). Three parallel samples were set up for each group, and the arithmetic mean value of the results was used as an error bar.

Fourier transform infrared spectroscopy (VERTEX 80V infrared spectrometer, Bruker, Karlsruhe, Germany) was used to study the gradient changes in its chemical properties. The bamboo slivers from L1 to L7 after tensile or bending mechanical experiments were obtained and tested. Bamboo powder with a size between 100 mesh and 200 mesh was dried at 105 °C, and then it was mixed with KBr in a ratio of 1:100 to obtain transparent ingot by a tablet press. Infrared spectroscopy scanning was performed in transmission mode, ranging from 500 cm^−1^ to 4000 cm^−1^, at a resolution of 4 cm^−1^, with 32 scans (Figure 3c).

An X-ray diffractometer (Ultima-IV Combined Multifunctional X-ray Diffractometer, Rigaku Co., Ltd., Tokyo, Japan) was used to test the crystallinity of bamboo. Bamboo powder with sizes between 150 mesh and 200 mesh from bamboo slivers (from L1 to L7) and bamboo strips was used. The test was carried out with the following parameters: Cu target Ka radiation, voltage 40 kV, current 30 mA. The scanning range was 5~80° (Figure 3d).

The relative crystallinity (CR) was calculated using the Segal method according to the intensity of the X-ray diffraction pattern [19]. Three parallel samples were set up in each group, and the arithmetic mean value of the results was used as an error bar. There was a maximum peak of (200) diffraction near the scanning curve 2θ ≈ 22.5°, and a minimum peak near 2θ ≈ 18°. The calculation formula for CR is:

$$CR=\frac{I_{200}-I_{am}}{I_{200}}\times 100\%$$

In the formula, CR represents the percentage of crystallinity, $I_{200}$ represents the maximum intensity of the (200) lattice diffraction angle, that is, the diffraction intensity of the crystalline region, and $I_{am}$ is the scattering intensity of the amorphous background diffraction at 2θ ≈ 18°.

## 3. Results and Discussion

### 3.1. Density and Vascular Bundle Distribution

Density is an important parameter to measure performance and influences the mechanical properties of bamboo [20].

**Figure 4:**
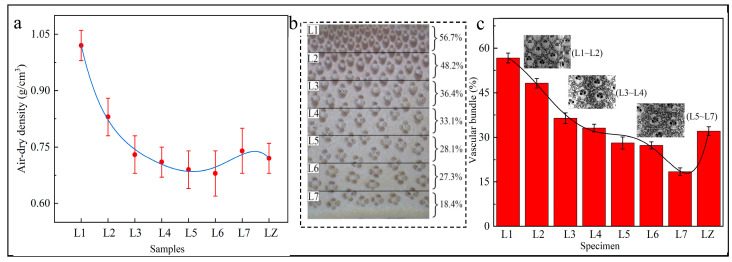
Density and vascular bundles distribution. (**a**) Air-dry density, (**b**) vascular bundle distribution, (**c**) proportion of vascular bundle.

The air-dry densities of L1 to L7 ranged from 0.68 g/cm^3^ to 1.02 g/cm^3^, with an average value of 0.77 g/cm^3^. From BGL to BYL, the air-dry density showed a decreasing and then increasing trend. This was mainly due to the fact that the BYL contained more cell wall material, which resulted in a density increase at the BYL (Figure 4a,b) [21].

We separated, extracted, and calculated the vascular bundles in the cross section of bamboo using the Image J software. The vascular bundle area percentage decreased from L1 to L7 (Figure 4b). Bamboo is a typical gradient material, with the vascular bundle distribution gradually becoming sparse from BGL to BYL (Figure 4c). This result was consistent with findings from other research, which found that the fiber–tissue ratio on the cross-section of bamboo decreases gradually along the radial direction of the bamboo wall [22,23].

### 3.2. Bending Property

**Figure 5:**
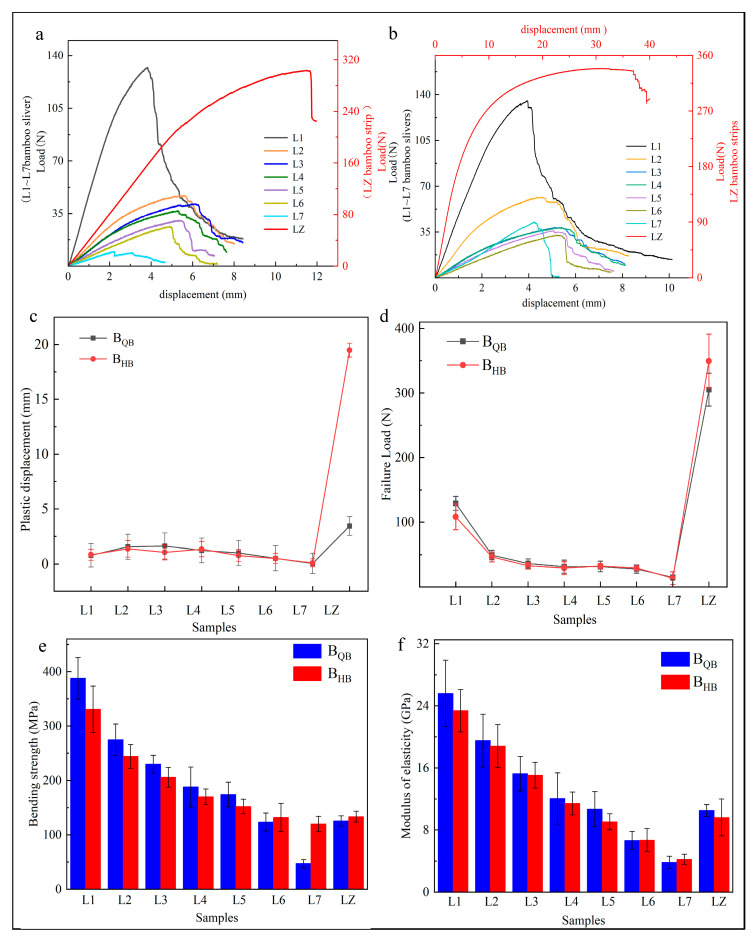
Mechanical properties of bending. (**a**) load–displacement curve of loading on bamboo green layer, (**b**) load–displacement curve of loading on bamboo yellow layer, (**c**) plastic displacement, (**d**) failure load, (**e**) bending strength, and (**f**) modulus of elasticity.

For the two bending directions, the bending load–displacement curves could each be divided into three stages. The first was the elastic phase, in which the load was linearly proportional to the displacement. After reaching the elastic proportional limit, it entered the plastic displacement phase until the bending load plummeted, i.e., it entered the failure stage (Figure 5a,b) [24]. The plastic displacements in the two loading directions both showed an M-shaped trend from L1 to L7, initially increasing, then decreasing, and increasing again.

The maximum displacement value was 1.63 mm and showed at L3 in the B_QB_ loading direction, while it was 1.34 mm and showed at L4 in the B_HB_ loading direction, both near the middle position along the radial bamboo wall. In both directions, the minimum plastic displacement was observed at the BYL L7 (Figure 5c), whose mechanical properties were significantly different from other bamboo slivers: After reaching the elastic proportionality limit, it entered the destructive stage with almost no plastic displacement (Figure 5a,b). The plastic displacement of bamboo strips in the two loading directions varied significantly, it was 5.6 times higher in the B_HB_ loading direction than in the B_QB_ loading direction (Figure 5c), indicating a substantial difference in deformation performance between these two directions.

The bending strength and modulus of elasticity both showed a decreasing trend from L1 to L7 on these two bending directions (Figure 5e,f). L1 was the highest, and L7 was the lowest. On the B_QB_ loading direction, the modulus of elasticity from L2 to L7 decreased by 23%, 21%, 20%, 11%, 37%, and 42%, respectively, compared to L1. In the B_HB_ loading direction, the modulus of elasticity decreased by 19%, 19%, 24%, 20%, 26%, and 36%, respectively, compared to L1. It showed the decrease in bending strength and modulus of elasticity near BYL were both more significant than other bamboo slivers.

The loading directions affected the bending property. In the B_QB_ loading direction, the bending modulus of elasticity of L1 (25.6 GPa) was 6.7 times that of L7 (3.8 GPa), and the bending strength of L1 (387.7 MPa) was 8.1 times that of L7 (47.3 MPa). The bending modulus of elasticity of the bamboo strip was 9.6 GPa, which is similar to L5, while the bending strength was 125.5 MPa, which is similar to L6. In the B_HB_ loading direction, the bending modulus of elasticity of L1 (23.3 GPa) was 5.5 times that of L7 (4.2 GPa), and the bending strength of L1 (330 MPa) was 3 times that of L7 (119.9 MPa). The bending modulus of elasticity of bamboo strip was 10.5 GPa, which is similar to L4, while the bending strength was 129.8 MPa, similar to L6 (Figure 5e,f). Furthermore, there is a significant difference in the failure load between layers L1 and L7 under both loading directions (Figure 5d). The change in chemical composition under the bamboo gradient structure is responsible for the great difference in the properties of L1 and L7.

Regardless of the loading direction, the bending properties near BGL were much better than those near BYL, and the difference was very significant, which was consistent with the results of Chen [25]. The bending toughness of bamboo was better when the bamboo green layer suffered tension stress [26]. The mechanical strength of bamboo strips close to the middle layer showed that the bamboo yellow layer was the weak layer in bamboo, which weakened the bending modulus of elasticity and strength [27]. One reason for this weakening effect of BYL was the gradient distribution of vascular bundle changes from bamboo outer layer to bamboo inner layer. The studies by Chen also indicated that the bending resistance decreased sequentially from BGL to BYL [28].

### 3.3. Tensile Property

**Figure 6:**
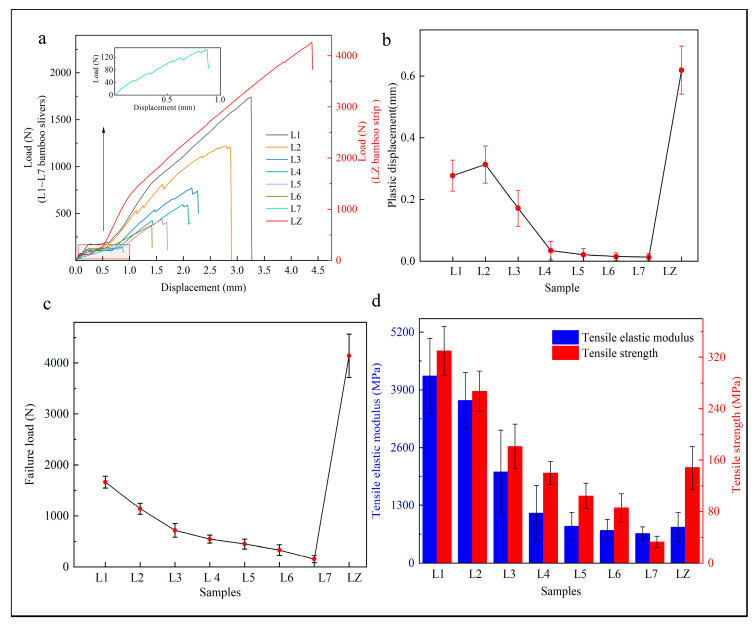
Mechanical properties of tensile. (**a**) load–displacement curve, (**b**) plastic displacement, (**c**) failure load, (**d**) tensile strength and modulus of elasticity.

The tensile load–displacement relationship curve also existed in three stages, from the elastic stage into the plastic displacement stage until the failure, and the closer to BGL, the more obvious of the three stages (Figure 6a). The plastic displacement from L1 to L7 increases first and then decreases, with L2 having the largest plastic displacement of 0.31 mm. The maximum plastic displacement of the bamboo strip is 0.61 mm, which is 1.9 times that of the bamboo strip, and the maximum plastic displacement of the bamboo strip occurs at L2 (Figure 6b).

The tensile failure loads from L1 to L7 are 1663.49 N, 1139.81 N, 715.88 N, 544.48 N, 449.88 N, 332.52 N, and 153.89 N, respectively, showing a general downward trend (Figure 6c). The difference between L1 and L7 is very large, the damage load of L1 is 10.4 times that of L7. The maximum failure load decreases rapidly from L1 to L3 (near the BGL) and slowly from L3 to L6 (around the BML).

From L1 to L7, the tensile strength and modulus of elasticity showed an overall trend of steady decrease (Figure 6d). The tensile strength for bamboo slivers from L1 to L7 and bamboo strips was 330.18 MPa, 267.28 MPa, 181.28 MPa, 139.92 MPa, 104.32 MPa, 85.68 MPa, 32.90 MPa, and 148.39 MPa, respectively. Compared with L1, it decreased by 19.09%, 32.17%, 22.81%, 25.44%, 17.86%, and 6.16% from L2 to L7. The tensile strength of L1 was the biggest and L7 the smallest, and L1 was 2.2 times larger than L7. A consistent conclusion was also found in Liu’s research, where tensile strength and modulus also decreased from the bamboo green layer to the bamboo yellow layer gradient [29].

A similar conclusion was also found in other research. Deng [30] measured the tensile strength and modulus of elasticity of bamboo slivers in different positions and found that the tensile strength showed an order of BGL > BML > BYL, which is consistent with this study. Huang et al. found that the tensile modulus of elasticity was the largest near the bamboo green layer and the smallest near the bamboo yellow layer, which showed a clear gradient-decreasing trend [6,31]. From the previous and this study, it is a universal conclusion that mechanical properties near the bamboo green layer are better than near bamboo yellow layer, whether the B_HB_ or B_QB_ direction of bending property [32].

### 3.4. Chemical Property

#### 3.4.1. Chemical Composition

**Figure 7:**
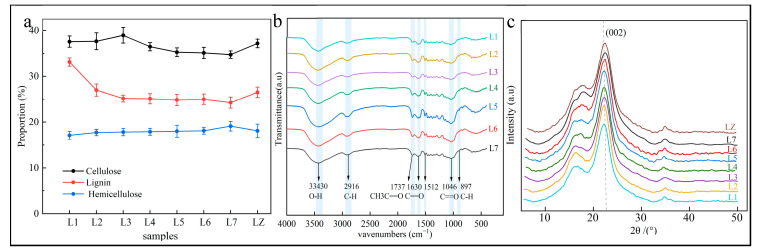
Chemical properties. (**a**) Chemical composition content, (**b**) FTIR spectra, (**c**) XRD spectra.

As a biomass material, bamboo is mainly composed of cellulose, hemicellulose, and lignin, and the chemical composition varies in different bamboo wall positions. From L1 to L7, the cellulose, hemicellulose, and lignin contents changed, and the change trend was different (Figure 7a). The cellulose and lignin contents decreased steadily, while the hemicellulose content showed a gradual, incremental trend. The hemicellulose content was highest at L3 38.96% and lowest at L7(34.75%). The lignin content was highest at L1 (33.12%) and lowest at L7(24.25%). The hemicellulose content profile was highest at L7 (18.44%) and lowest at L1 (17.31%). The lignin content of L7 decreased by 26.78%, and the hemicellulose content increased by 6.12% compared to L1. Han [8] found that the functional gradient largely determined the physical properties of the bamboo layer, and the gradient distribution of chemical composition contributed to the functional gradient of bamboo.

The Fourier transform infrared spectroscopy (FTIR) analysis technique was an effective tool to study the chemical functional group gradient change of bamboo [33]. From the FTIR spectra, it could be seen that the intensities and positions of the main absorption peaks in different bamboo slivers were basically the same, which indicates that the bamboo slivers have the same chemical composition (Figure 7b). There is a distinct characteristic peak at wave number 3343 cm^−1^, which originated from the hydroxyl stretching vibration [34] and is the key functional group influencing the dimensional stability of bamboo. The absorption peak at 2916 cm^−1^ was a C–H stretching vibration absorption peak, which is a typical cellulose characteristic peak. The C=O stretching vibration peaks near 1737 cm^−1^ and near 1046 cm^−1^ are characteristic peaks to characterize hemicellulose. The absorption peaks generated by the vibration of the benzene ring carbon skeleton near 1594 cm^−1^ and 1630 cm^−1^ could be used to characterize lignin [35]. Near 1512 cm^−1^, there is an absorption peak attributed to the stretching vibration of lignin, representing the aromatic skeleton vibration in lignin [36]. The 897 cm^−1^ spectral band was the characteristic peak of the β-gluconic anhydride bond and the characteristic absorption peak of the cellulose C–H bending vibration [33]. The results of FTIR spectroscopic testing of bamboo slivers were consistent with the results of chemical composition content testing.

#### 3.4.2. Relative Crystallinity

From the XRD spectra, it could be seen that there were diffraction peaks near 16.0° and 22°, with the strongest diffraction peaks occurring at 22° (Figure 7c). This diffraction peak is attributed to the characteristic peaks of cellulose I [37]. The shapes of the diffraction intensity curves of bamboo slivers were basically the same, indicating that the cellular structure of each gradient layer has not been changed.

The CR has slightly changed (Table 1), mainly showing a decreasing trend from BGL to BYL. The CR showed the highest at L2 and the lowest at L7, which was supposed to be due to the high cellulose content and low hemicellulose and lignin content of L2 (Figure 7a). The crystallinity of cellulose was defined as the percentage of the crystalline region in cellulose [38]; the higher the cellulose content, the higher the CR of bamboo [39]. The larger the CR, the stronger the intermolecular bonding ability; the tensile strength, bending strength, and dimensional stability would also be increased, which is consistent with this study (Figure 5e,f and Figure 6d).

## 4. Correlation Analysis

**Figure 8:**
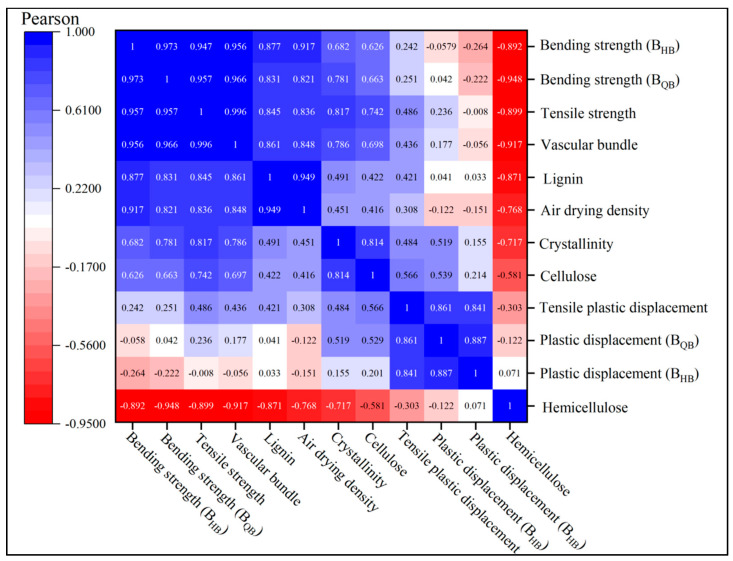
Correlation coefficient of the testing factors.

Firstly, SPSS software (R27.0, IBM, Chicago, IL, USA) is used to perform correlation calculations about the above parameters and present the relationships between mechanical, physical, and chemical properties using a correlation heatmap (Figure 8). From the figure, it can be observed that the bending and tensile strength of bamboo are primarily influenced by individual factors such as vascular bundle percentage, hemicellulose content, and lignin content. The order of influence on the tensile strength of bamboo is as follows: vascular bundle percentage > hemicellulose content > lignin content > density > CR.

The vascular bundle is the primary load-bearing structural unit of bamboo and has a significant impact on the mechanical properties of bamboo. The vascular bundles near the bamboo green layer are small and densely distributed, while the bamboo yellow layer is large and sparse distributed, and the load ability of bamboo changes with the vascular bundle [40]. There is a strong positive correlation between vascular bundle area percentage and the mechanical properties of bamboo: the correlation coefficients are 0.956 and 0.966 for B_HB_ and B_HQ_, respectively, and 0.996 for tensile strength.

The lignin content shows strong positive correlation with the mechanical properties of bamboo; the correlation coefficients for _BHB_ and B_HQ_ are 0.877 and 0.831, respectively, and that for tensile strength 0.845. This occurs because lignin, a vital constituent of the cell wall, frequently permeates between cellulose and hemicellulose, contributing to the enhancement of bamboo’s mechanical properties [41].

The density of bamboo is an indication of the amount of material per unit volume and is closely related to its mechanical properties [42]. The air-dry density showed a strong positive correlation with the mechanical properties of bamboo. The correlation coefficients of air-dry density for B_HB_ and B_HQ_ were 0.917 and 0.821, respectively, and the correlation coefficient for tensile strength was 0.836.

Relative crystallinity showed a moderate positive correlation with B_HB_ and a strong positive correlation with B_HQ_, with correlation coefficients of 0.682 and 0.781, respectively, and a positive correlation with tensile strength, with a correlation coefficient of 0.817. This is because a higher relative crystallinity makes the intermolecular arrangement tightly ordered; the porosity decreases, the intermolecular interaction force increases, and the mechanical properties improve [43].

Furthermore, Figure 8 also shows a strong negative correlation between hemicellulose content and mechanical properties of bamboo, and the correlation coefficients between hemicellulose and B_HB_ and B_HQ_ were −0.892 and −0.948, respectively, and the correlation coefficient for tensile strength was −0.899. This is due to the fact that hemicellulose is hydrophilic in nature, and excessive levels can lead to reduced strength [44].

The percentage of vascular bundles plays a decisive role in the mechanical properties of bamboo [45], and the gradient distribution of vascular bundles along the direction of bamboo diameter is the main reason that causes the gradient decrease in bamboo’s mechanical strength from BGL to BYL [46].

The correlation relationship between bending strength and vascular bundle, tensile strength and vascular bundle, bending strength and density, and tensile strength and density were explored further. It was found that vascular bundle area percentage and density were both linearly correlated with mechanical properties (*p* < 0.05), while lignin content and CR were curve-linearly correlated with mechanical properties (*p* < 0.05).

**Figure 9:**
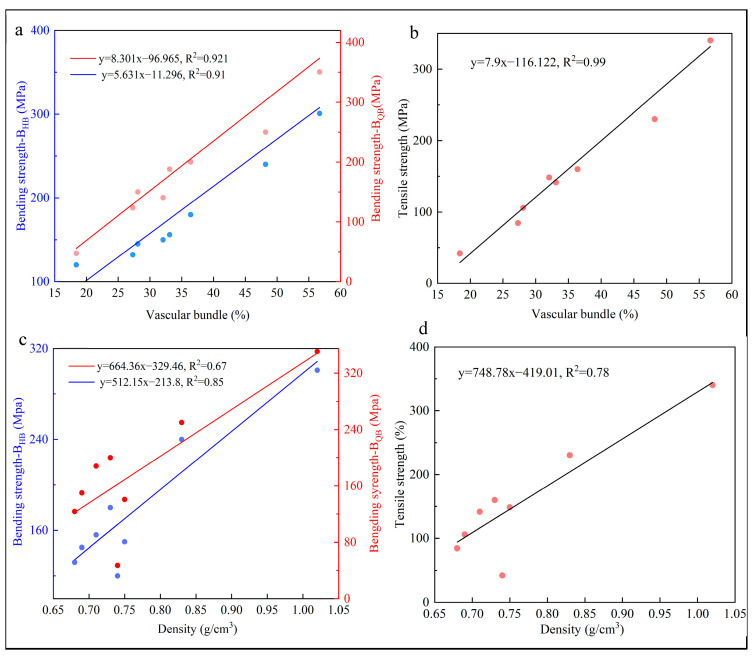
Correlation between microstructure and mechanical properties. (**a**) Vascular bundle and bending strength on B_QB_ and B_HB_, (**b**) vascular bundle and tensile strength, (**c**) density and bending strength on B_QB_ and B_HB_, (**d**) density and tensile strength.

The vascular bundle area percentage showed a positive linear relationship with bending strength and tensile strength, with R^2^ above 0.9 in all cases (Figure 9a,b), which verifies that vascular bundles have a significant reinforcing effect on the mechanical properties of bamboo [47]. There was a positively linear relationship between density and bending strength and tensile strength, with R^2^ ranging from 0.67 to 0.78 (Figure 9c,d). In addition to the vascular bundle area percentage, density played a secondary and important role in the mechanical properties of bamboo [48].

**Figure 10:**
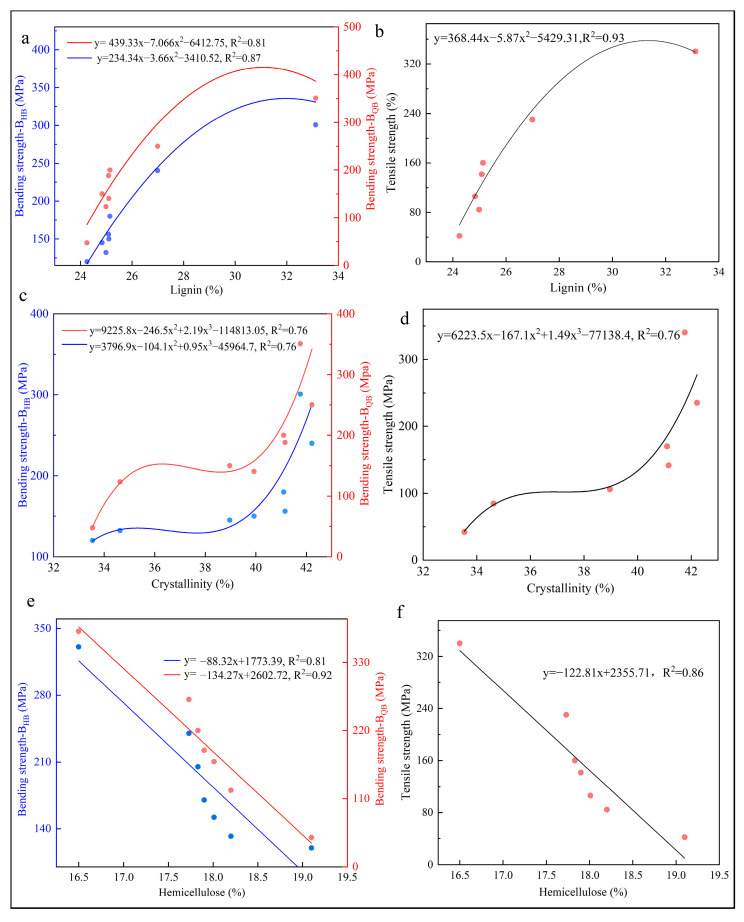
Correlation relationship between chemical composition and mechanical properties (**a**) lignin and bending strength on B_QB_ and B_HB_, (**b**) lignin and tensile strength, (**c**) crystallinity and bending strength on B_QB_ and B_HB_, (**d**) crystallinity and tensile strength, (**e**) hemicellulose and bending strength, (**f**) hemicellulose and tensile strength.

Concerning the bending strength on B_HB_, B_QB_ and the lignin content, and the tensile strength and the lignin content, a well-parabolic-linear correlation is obtained, with R^2^ above 0.8 (Figure 10a,b). Lignin, as one of the major components of the cell wall, enhances the connection between cells and improves the mechanical properties of bamboo. It has been shown that differences in the distribution pattern and structure of lignin can lead to large differences in the physical-mechanical and chemical properties of bamboo, which suggests that lignin chemical composition directional cultivation during bamboo growth would be useful for the processing and utilization of bamboo [49]. There is a turning point in the lignin content that affects bending strength and tensile strength. When the lignin content values fall within the range of 24% to 28%, the growth rates of bending strength for B_HB_, bending strength for B_QB_, and tensile strength increase with the increase in lignin content. After the lignin content exceeds 28%, the enhancing effect on mechanical properties begins to weaken.

For CR to bending strength on B_HB_, B_QB_, and tensile strength, a well-positive correlation is obtained with R^2^ around 0.76 in all cases (Figure 10c,d). As the crystallinity content increases, the bending strength on B_HB_, B_QB_, and tensile strength all increase sequentially. There was a turning point in the CR of around 35% on bending strength and tensile strength. When the CR reached 35%, the bending strength on B_HB_, B_QB_, and tensile strength grew slowly or even decreased. While CR was higher than 39%, the growth rate became faster, which indicated that CR is an important factor affecting the mechanical properties of bamboo and provides mechanical strength for bamboo [50]. One of the reasons for crystallinity change is that the number of fiber cells increases from the bamboo green layer to the bamboo yellow layer, which leads to a higher cellulose content in the bamboo green layer than the bamboo yellow layer [51]. Moreover, the most important role of cellulose is to increase the strength and stiffness of bamboo. Therefore, as the proportion of the cellulose crystalline zone in the bamboo yellow layer decreased, the mechanical properties of bamboo also decreased.

There was a significant negative linear relationship between hemicellulose content and bending, tensile strength (Figure 10e,f), with R^2^ ranging from 0.8 to 0.92. The hemicellulose content of bamboo decreases from BGL to BYL. This is because hemicellulose is hydrophilic, which can enhance the flexibility of bamboo to a certain extent, but an excessive amount can affect its strength negatively [44].

However, if the hemicellulose content was too high, it would lead to a decrease in the hardness and strength of bamboo [52]. The bending and tensile strength of bamboo gradually decreased with the increase in hemicellulose content in this experiment.

## 5. Conclusions

Moso bamboo is a typical multi-gradient natural material, and its gradient structure has a significant effect on mechanical properties. The mechanical properties of bamboo are influenced by physical and chemical properties in the following order: Vascular bundle area percentage, hemicellulose content, lignin content, density, and relative crystallinity. The gradient distribution of vascular bundle area percentage is the main reason for bamboo mechanical strength decreasing in the bamboo radial direction.

Vascular bundle area percentage and density exhibited a linear and positive correlation with mechanical properties, whereas hemicellulose content showed a linear and negative correlation with mechanical properties.

With an increase in lignin content and crystallinity, the bending strength and tensile strength of bamboo increased. However, with lignin content surpassing 28%, there was a deceleration in the enhancement of both bending and tensile strength in bamboo. Likewise, upon reaching a relative crystallinity of 35%, there was either a slowdown or even a decline in the improvement of bending and tensile strength in bamboo. The variations in chemical content and characterization parameters influence its tensile and flexural mechanical properties.

Bamboo, with its high strength and exceptional mechanical properties, enjoys widespread utilization in construction, home furnishing, and diverse industries. However, the evident structure gradient characteristics introduce pronounced variations in density, mechanics, and dimension stability. The gradient shifts in performance have not been adequately addressed in industrial applications, consequently limiting the quality and service life of bamboo products. This study elucidates the interrelations among the physical, chemical, and mechanical properties of bamboo, offering insights to enhance the processing efficiency of moso bamboo and optimize product structures. Such insights would facilitate informed choices in bamboo material selection for practical applications. Future research endeavors should prioritize refining processing techniques and exploring innovative industrial applications for bamboo.

## Figures and Tables

**Table 1:** The relative crystallinity (CR) of bamboo slivers and bamboo strips.

Samples	L1	L2	L3	L4	L5	L6	L7	LZ
CR	41.77%	42.22%	41.1%	41.16%	38.97%	34.63%	33.54%	39.93%
SD	±1.22%	±0.85%	±0.97%	±0.76%	±0.79%	±0.91%	±0.83%	±0.64%

## Data Availability

Data are contained within the article.
